# A Letter to Dr. Chambers, on the Nature and Proper Treatment of Gout

**Published:** 1839-04

**Authors:** 


Art. IX.—*A Letter to* Dr. Chambers, *on several important Points relating to the Nature and proper Treatment of Gout.* By Sir Charles Scudamore, m.d. f.r.s. &c.—*London,* 1839. 8vo. pp. 59.

Although Sir Charles Scudamore tells us, at the conclusion of this letter, that he has "brought forward many new arguments and new facts and observations, with the design of elucidating the real nature of gout;" and "indulges in the hope" that he has "successfully advocated the pretensions of regular practice, in opposition to empirical methods of treatment;" we must confess our inability, after a careful perusal of the pamphlet, to discover any trace of such facts, or any rational grounds for the indulgence of such hope. They who have read the author's elaborate treatise on Gout will, we think, find nothing new in the Letter; and they who read the letter only will derive little benefit from it, unless it lead them to peruse the treatise. The whole is a rambling, gossiping, ill-written, and ill-arranged compilation from the author's former works, calculated rather to detract from his own reputation, and capable of doing no manner of good, unless it should chance to fall into the hands of some ponderous dowager or plethoric alderman, who might be moved by its eloquent appeals to eschew the dangers of self-treatment and the high-pressure power of colchicum, and submit themselves to rational medication in the hands of Sir Charles himself. Here, we admit, the gain to them would be great; for it is clear that his treatment is on the whole good, although in no respect, that we can discover, peculiar, unless in the continued advocacy of the *acetum colchici,* in preference to the stronger preparations. It is curious, however, that the author should have omitted to state the precise formula or dose of his favorite remedy in a work, the main object of which seems to be to recommend it. At p. 37 he says, "I am led both by theory and experience to prescribe the mildest preparations of colchicum, and those in combination with saline aperients; using this medicine as a valuable palliative for a short period during the existence of the gouty irritation, but not viewing it as the really curative agent. The draught to which I alluded at p. 6," &c. On turning back to p. 6, we read, "With respect to the particular preparation, I have made choice of the acetum, which is much weaker than the wine; this last being prepared with about twelve times the quantity of colchicum; besides that, the acetic acid modifies the principles of the root (?), and gives it a milder operation. I found advantage from joining it with magnesia and sulphate of magnesia; and thus directing its action to the bowels and the kidneys, and using with the vehicle of the ingredients some aromatic water or tincture, it rarely happened that the stomach itself was disordered by the repetition of the dose." And this is the nearest approach we make to the knowledge of the dose of the remedy or the composition of *the draught.* We trust that Sir Charles Scudamore may at least have the satisfaction of having his work looked graciously down on by his friend Dr. Chambers, from "the highest pinnacle of the profession;" and should the College of Physicians, in their new-born zeal for radical reform and medical emancipation, throw their gates still wider for the entrance of licentiates, we hope that the learned inditer of the Letter will not be forgotten, nor the *recherché* compliment emblazoned on the title-page from the writings of that great author Cicero Medicus.

